# Characterizing New England Emergency Departments by Telemedicine Use

**DOI:** 10.5811/westjem.2017.8.34880

**Published:** 2017-09-11

**Authors:** Kori S. Zachrison, Emily M. Hayden, Lee H. Schwamm, Janice A. Espinola, Ashley F. Sullivan, Krislyn M. Boggs, Ali S. Raja, Carlos A. Camargo

**Affiliations:** * Massachusetts General Hospital, Department of Emergency Medicine, Boston, Massachusetts; † Massachusetts General Hospital, Department of Neurology, Boston, Massachusetts

## Abstract

**Introduction:**

Telemedicine connects emergency departments (ED) with resources necessary for patient care; its use has not been characterized nationally, or even regionally. Our primary objective was to describe the prevalence of telemedicine use in New England EDs and the clinical applications of use. Secondarily, we aimed to determine if telemedicine use was associated with consultant availability and to identify ED characteristics associated with telemedicine use.

**Methods:**

We analyzed data from the National Emergency Department Inventory-New England survey, which assessed basic ED characteristics in 2014. The survey queried directors of every ED (n=195) in the six New England states (excluding federal hospitals and college infirmaries). Descriptive statistics characterized ED telemedicine use; multivariable logistic regression identified independent predictors of use.

**Results:**

Of the 169 responding EDs (87% response rate), 82 (49%) reported using telemedicine. Telemedicine EDs were more likely to be rural (18% of users vs. 7% of non-users, p=0.03); less likely to be academic (1% of users vs. 11% of non-users, p=0.01); and less likely to have 24/7 access to neurology (p<0.001), neurosurgery (p<0.001), orthopedics (p=0.01), plastic surgery (p=0.01), psychiatry (p<0.001), and hand surgery (p<0.001) consultants. Neuro/stroke (68%), pediatrics (11%), psychiatry (11%), and trauma (10%) were the most commonly reported applications. On multivariable analysis, telemedicine was more likely in rural EDs (odds ratio [OR] 4.39, 95% confidence interval [CI] 1.30–14.86), and less likely in EDs with 24/7 neurologist availability (OR 0.21, 95% CI [0.09–0.49]), and annual volume <20,000 (OR 0.24, 95% CI [0.08–0.68]).

**Conclusion:**

Telemedicine is commonly used in New England EDs. In 2014, use was more common among rural EDs and EDs with limited neurology consultant availability. In contrast, telemedicine use was less common among very low-volume EDs.

## INTRODUCTION

Resource availability in U.S. emergency departments (EDs) varies substantially, particularly in rural areas with disparities in access^1^ and in smaller EDs reporting decreased consultant availability.^2^ Telemedicine (TM), the use of telecommunication for remote diagnosis or treatment, may be part of the solution to connect patients with the resources necessary for their care.^3^ It is feasible and effective for clinical care in EDs,^4^ may improve care coordination,^1^ and its value has been well-established in emergency stroke care.^5^

The promise of TM has been underscored by the 21^st^ Century Cures Act and the Expanding Capacity for Health Outcomes Act.^6^,^7^ Yet the extent of TM adoption in U.S. EDs is not well known. As a first step, our primary objective was to describe the prevalence of TM use in New England EDs and the applications for which it is used. Secondarily, we aimed to identify independent predictors of ED TM use.

## METHODS

### Study Design, Setting and Population

We conducted a survey of New England EDs as part of the National Emergency Department Inventory (NEDI). This institutional review board-approved study, called NEDI-NE, was coordinated by the Emergency Medicine Network.^8^ We used the NEDI-USA 2012 database to obtain a comprehensive list of all EDs in New England (Connecticut, Maine, Massachusetts, New Hampshire, Rhode Island, Vermont); the methods that underlie NEDI-USA have been reported.^9^ Briefly, EDs are included if they are open 24/7 and available for use by the general public. This includes hospital-based EDs, and hospital-affiliated freestanding EDs. We excluded EDs at federal hospitals and college infirmaries.

### Survey and Administration

We administered the survey (Appendix 1) in 2015, with modules to characterize the EDs in 2014 including basic characteristics, staffing, electronic resources, and timing of consultations. The survey content and wording were refined with feedback from colleagues, including the Massachusetts College of Emergency Physicians’ Board of Directors, which includes both community and academic physicians. We mailed surveys to ED directors up to three times over a two-month period; a link to an online-version of the survey was included in each mailing. Follow-up to non-responsive sites, and those with partially completed surveys, was conducted through telephone calls and site visits. We entered and managed survey data using the REDCap electronic data capture tool.

### Measurements

Our primary outcome was use of TM. For this study, we focused on the use of video consultation for patient evaluation, which excludes store-and-forward technology (e.g., teleradiology or dermatology). We determined TM use based on the survey item, “Does your ED obtain consultation via video conferencing equipment? (e.g., video transmission to outside experts for evaluation of an acute stroke patient in your ED)?” Respondents who selected “yes” received a free-text field to “specify” the type of consultation.

**Population Health Research Capsule**

What do we already know about this issue?

Telemedicine is a feasible and effective technology to use in EDs for remote diagnosis, treatment, and care coordination.

What was the research question?

What is the prevalence of telemedicine use in New England EDs and what are the applications for which it is used?

What was the major finding of the study?

About half of New England EDs report using telemedicine, most commonly for neuro/stroke, pediatrics, psychiatry, and trauma.

How does this improve population health?

Telemedicine may be a means to address disparities in access for patients in rural or underserved areas. These results begin to lay the groundwork for future research in emergency telemedicine.

We also collected data regarding other key ED characteristics related to staffing, patient volume, bed size, and availability of specialists for consultation. We categorized EDs by annual volume (<20,000, 20,000–39,999, 40,000–59,999, and ≥60,000 visits per year). We classified EDs as urban or rural based on location in a core-based statistical area.^9^ We defined academic EDs as any site affiliated with an emergency medicine residency program designated by the Society for Academic Emergency Medicine.^10^

### Data Analysis

Descriptive statistics quantified ED TM use, and applications of its use are presented as proportions and medians (with interquartile ranges [IQR]). To better understand the relationship between TM use and multiple ED characteristics, we performed bivariate analyses using chi-square test, Fisher’s exact test, and Wilcoxon rank-sum test, as appropriate. To identify independent predictors of TM use among New England EDs, we used multivariable logistic regression. Model covariates were specified *a priori*; given absence of literature on TM use in EDs, variables were selected based on our hypotheses and clinical experience. The final model adjusted for rural location, annual ED visit volume, percentage of uninsured/self-pay patients, number of critical care transfers, number of full-time attending physicians, and neurology consultant availability. Results are reported as odds ratios (ORs) with 95% confidence intervals (CI). We performed analyses with Stata 14.1 (Stata Corp, College Station, TX).

## RESULTS

### ED Responses and Characteristics

Of the 195 New England EDs surveyed, we received responses from 169 (87%). Responding and non-responding EDs were similar on several important variables (e.g., rural location, academic status, annual visit volume: Appendix 2). Among all responding EDs, 12% were rural and 7% academic (Table). The median number of ED visits in 2014 was 30,000 (IQR 16,000–51,000). The median number of ED beds was 22 (IQR 11–33).

### Telemedicine Use in New England EDs

Of the 169 responding EDs, 82 (49%) reported using TM. The most commonly reported applications were stroke/neuro, pediatrics, psychiatry, and trauma (Figure). In bivariate analyses examining the association between TM use and ED characteristics (Table), TM-using EDs were more often rural and less often academic. TM-using EDs had a lower median annual ED volume, but did not vary by annual total children ED visits. TM-using EDs had fewer beds, reported fewer full-time attending physicians, and less frequently reported a 24/7 certified emergency nurse on duty, but there was no difference in the proportion of EDs with 24/7 attending physician coverage. Relative to EDs without TM, TM-using EDs had more critical care transfers during the study year.

TM-using EDs had less availability of consultants for some, but not all, specialties (Table). For example, 30% of TM-users reported 24/7 neurology availability, versus 63% of non-users. Compared to non-users, TM-users had less availability of neurologists, neurosurgeons, plastic surgeons, psychiatrists, hand-surgeons, and orthopedic surgeons.

### Predictors of ED Telemedicine Use in Multivariable Model

In multivariable logistic regression modeling, rural EDs were more likely to use TM (OR 4.39 95% CI [1.30–14.86]), and EDs with 24/7 neurologist availability were less likely to use TM (OR 0.21, 95% CI [0.09–0.49]). Relative to EDs with an intermediate number of visits, smaller EDs (i.e., less than 20,000 annual visits) were less likely to use TM (OR 0.24, 95% CI [0.08–0.68]).

## DISCUSSION

In this study, we found that nearly half of New England EDs use TM. The most commonly reported applications were stroke/neurology, pediatrics, psychiatry, and trauma. In multivariable modeling, ED TM use was associated with rural location, lack of 24/7 neurologist availability, and annual visit volume of 20,000 or more.

The relationship between annual ED volume and TM use warrants further consideration, and may be a consequence of the expense required for TM implementation. We do not believe that this finding was influenced by the association between rural location and TM use, as the relationship between volume and TM use did not significantly change when rural location was removed from the regression model (Appendix 3).

It is not surprising that stroke and pediatrics were two of the four most frequently reported applications, given substantial bodies of literature for the use of TM to improve acute stroke care delivery^5^ and pediatric critical care.^11^ TM has many other potential applications in EDs, for example, to augment care provided in EDs staffed by nurse practitioners and physician assistants, to reduce patient transfers, and even to maintain 24/7 ED staffing.^12^–^15^ TM may also enable increased access to emergency psychiatry services^16^ – an application of tremendous potential value to U.S. EDs that are strained by psychiatric emergencies and patient boarding.^17^

While the use of TM for particular applications in emergency medicine has been studied, we are not aware of any prior descriptions of the prevalence of TM use in U.S. EDs. This regional description is a valuable first step. Future work is needed to describe TM use in EDs nationally, to understand barriers and facilitators of TM implementation, to evaluate safety of TM, and to consider policy changes that may motivate adoption, in particular related to reimbursement for TM services.

## LIMITATIONS

One potential limitation of this work is related to sampling. Of 195 New England EDs, 169 surveys were completed; this non-response could have influenced results. However, we believe the 87% response rate is sufficient to characterize TM use among New England EDs and do not have any reason to expect response bias with this particular question. Likewise, we do not have reason to believe that non-responders are systematically different in TM use than responders, as the primary focus of the survey was not on TM use, and we did not find differences between the groups in rural location or annual visit volume.

Secondly, this description of TM use in New England EDs may not be generalizable. New England has distinct characteristics that may influence our findings, such as closer distances between hospitals and fewer rural hospitals than some other U.S. regions; our definition of rural EDs may not accurately reflect distance from a referral hospital. Additionally, New England may have differences in the political and economic environment surrounding TM.

Another limitation is with respect to the outcome. We chose to focus on video conferencing for patient evaluation. Therefore, our findings are unlikely to reflect the more commoditized forms of TM applications such as teleradiology. Finally, given the nature of our survey data we were unable to extract further information from EDs to characterize the nature of TM use or to explore explanatory models for our findings. Nevertheless, we believe these findings provide important preliminary results to inform future study of ED TM use.

## CONCLUSION

Telemedicine is used in nearly half of New England EDs. It is more often used in EDs that are rural, EDs that do not have 24/7 neurologist availability, and EDs with annual volume of greater than or equal to 20,000 visits. TM may have value beyond its current applications in emergency medicine, such as for workforce and resource-related issues. As TM becomes more prominent in U.S. healthcare policy,^6^,^7^ future research should characterize TM use in EDs nationally, as well as barriers and facilitators of its implementation.

## Supplementary Information







## Figures and Tables

**Figure:**
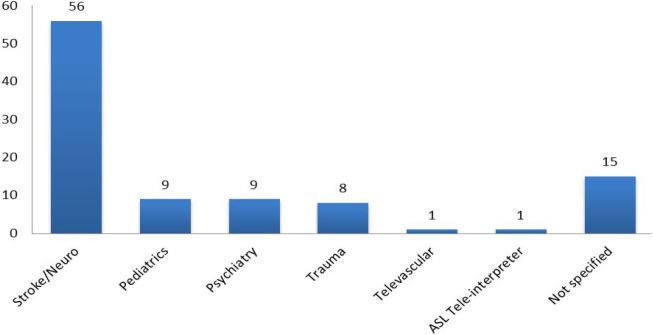
Free responses as reported by responding EDs converted to categorical variable. Type of telemedicine (TM) use reported by TM-using EDs, n=82.

**Table:** New England emergency department characteristics by telemedicine use.

ED characteristics	Telemedicine non-users n=87	Telemedicine users n=82	p-value
Rural*†	6 (7)	15 (18)	0.03
Academic ED†	10 (11)	1 (1)	0.01
Freestanding ED†	2 (2)	3 (4)	0.60
Median annual total ED visits (IQR)	35,126 (17,500–59,112)	26,730 (14,925–40,000)	0.02
Annual total ED visits			0.03
<20,000	25 (29)	29 (35)	
20,000–39,999	22 (25)	31 (38)	
40,000–59,999	21 (24)	16 (20)	
≥60,000	19 (22)	6 (7)	
Median annual total ED visits by children (IQR)	3,600 (1,500–8,395)	3,425 (2,0005,000)	0.42
Median number of ED beds (IQR)	25 (13–39)	20 (9–29)	0.01
Percentage of uninsured/self-pay			0.25
<10%	30 (34)	35 (43)	
≥10%	46 (53)	33 (40)	
Unknown	11 (13)	14 (17)	
Number of critical care transfers			0.02
<250	64 (74)	43 (52)	
≥250	18 (21)	31 (38)	
Unknown	5 (6)	8 (10)	
Median number of full-time attending physicians (IQR)	11 (6–22)	9 (5–13)	0.04
24/7 Attending Physician on duty			1.00
No	5 (6)	5 (6)	
Yes	82 (94)	77 (94)	
24/7 Certified emergency nurse on duty			0.046
No	19 (23)	32 (40)	
Yes	52 (62)	36 (45)	
Don’t know	13 (15)	12 (15)	
Specialist availability			
Anesthesiologist			
in-person	77 (89)	71 (87)	0.71
24/7	72 (83)	69 (84)	0.81
Cardiologist			
in-person	67 (77)	62 (76)	0.83
24/7	59 (68)	48 (59)	0.21
General surgeon			
in-person	79 (91)	77 (94)	0.45
24/7	76 (87)	65 (79)	0.16
Neurologist			
in-person	63 (72)	40 (49)	0.002
24/7	55 (63)	25 (30)	<0.001
Neurosurgeon			
in-person	39 (45)	16 (20)	<0.001
24/7	35 (40)	10 (12)	<0.001
Obstetrician/ gynecologist			
in-person	74 (85)	64 (78)	0.24
24/7	72 (83)	60 (73)	0.13
Orthopedist			
in-person	79 (91)	69 (84)	0.19
24/7	70 (80)	51 (62)	0.009
Pediatrician			
in-person	56 (64)	56 (68)	0.59
24/7	50 (57)	48 (59)	0.89
Plastic surgeon			
in-person	41 (47)	22 (27)	0.01
24/7	24 (28)	9 (11)	0.01
Psychiatrist			
in-person	54 (62)	35 (43)	0.01
24/7	40 (46)	16 (20)	<0.001
Hand Surgeon			
in-person	51 (59)	28 (34)	0.001
24/7	31 (36)	10 (12)	<0.001

*ED*, emergency department; *CBSA*, core-based statistical area; *IQR*, interquartile range; *24/7,* available 24 hours a day and 7 days a week.

Data are no. (%) of EDs unless otherwise indicated. Percentages may not total to 100% due to rounding.

* Defined by location outside of a core-based statistical area.

† Acquired from 2013 NEDI-USA.

*24/7,* available 24 hours a day and 7 days a week.
