# Supplementary material for: Characterizing New England Emergency Departments by Telemedicine Use
Source: West J Emerg Med. 2017 Sep 11;18(6):1055–60. doi: 10.5811/westjem.2017.8.34880 (PMC5654874; doi:10.5811/westjem.2017.8.34880)

**APPENDIX 1.** Survey Instrument (Note: a distinct version was created for each state in the region. The Massachusetts version of the survey is presented here as an example).


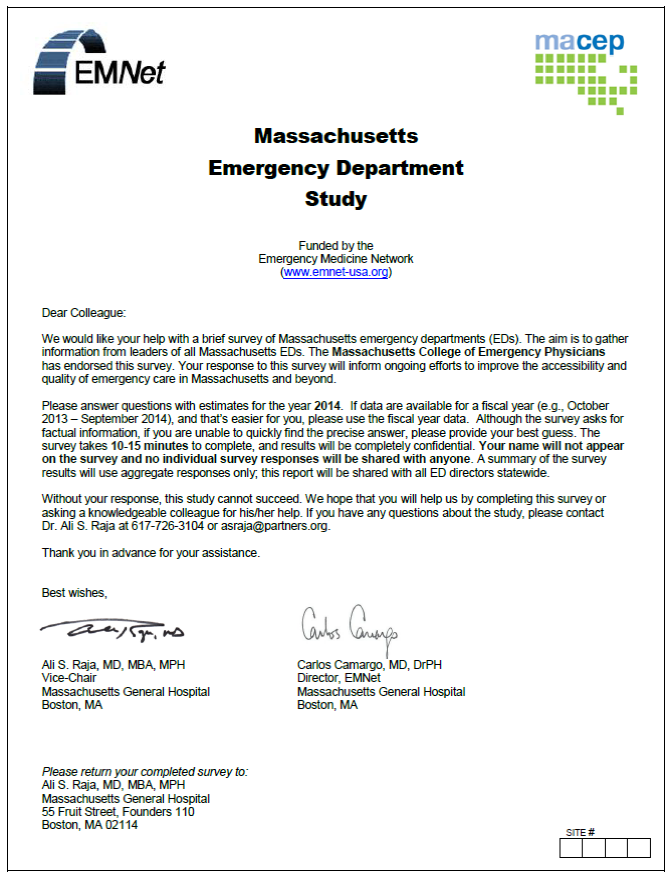


**
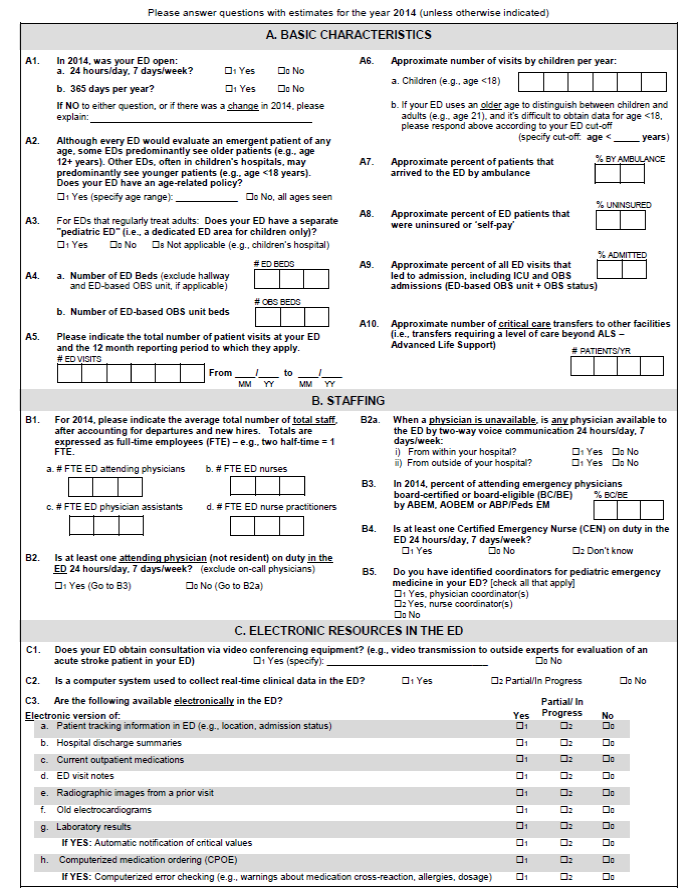
**

**
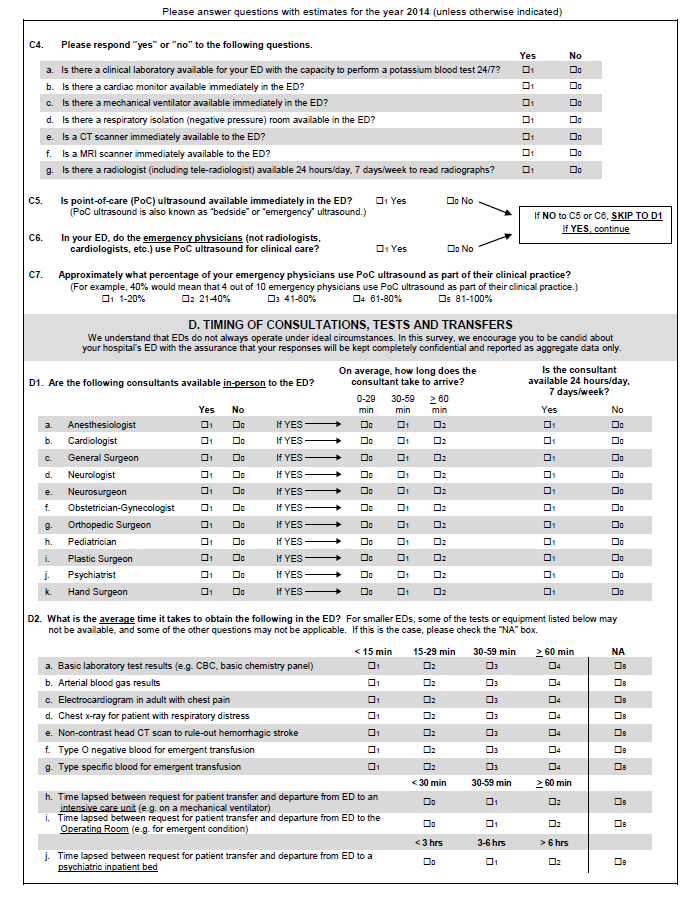
**


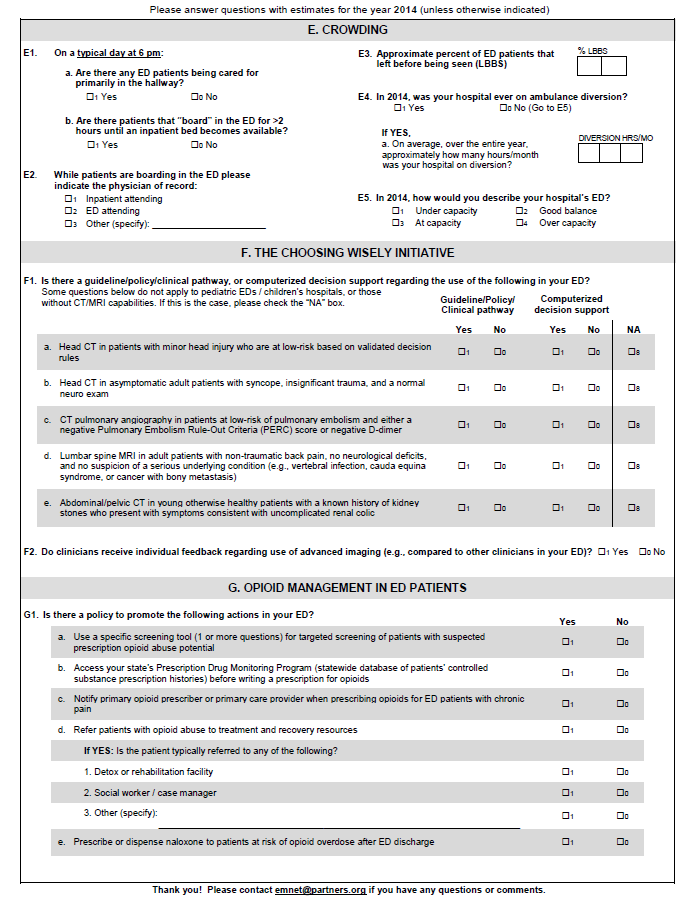

Supplement: Supplementary file 1 [file wjem-18-1055-s001.docx]
