# Supplementary material for: Characterizing New England Emergency Departments by Telemedicine Use
Source: West J Emerg Med. 2017 Sep 11;18(6):1055–60. doi: 10.5811/westjem.2017.8.34880 (PMC5654874; doi:10.5811/westjem.2017.8.34880)
Supplement: Supplementary file 2 [file wjem-18-1055-s002.docx]

**APPENDIX 2**. Table A2. Comparison of Responding vs. Non-Responding EDs

| **Characteristics** | **Non-responders(n=26)** | **Responders (n=169)** |  |
| --- | --- | --- | --- |
|  | ***n (row %)*** | | **P-value** |
| **State** |  |  | 0.99 |
| CT | 4 (11%) | 31 (89%) |  |
| MA | 10 (14%) | 63(86%) |  |
| ME | 6 (17%) | 30 (83%) |  |
| NH | 3 (12%) | 23 (88%) |  |
| RI | 1 (9%) | 10 (91%) |  |
| VT | 2 (14%) | 12 (86%) |  |
| **Hospital located in CBSA** |  |  | 0.07 |
| No | 7 (25%) | 21 (75%) |  |
| Yes | 19 (11%) | 148 (89%) |  |
| **Academic ED** |  |  | 0.37 |
| No | 26 (14%) | 158 (86%) |  |
| Yes | 0 (0%) | 11 (100%) |  |
| **Freestanding ED** |  |  | 0.58 |
| No | 25 (13%) | 164 (87%) |  |
| Yes | 1 (17%) | 5 (83%) |  |
| **Median Visit Volume in 2013 (IQR)** | 24,143 (15,219-39,311) | 32,000 (17,000-54,984) | 0.17 |

Data based on 2013 NEDI-USA survey.
