# Supplementary material for: Characterizing New England Emergency Departments by Telemedicine Use
Source: West J Emerg Med. 2017 Sep 11;18(6):1055–60. doi: 10.5811/westjem.2017.8.34880 (PMC5654874; doi:10.5811/westjem.2017.8.34880)

**APPENDIX 3.** Examining the Contribution of Rural Hospital Status

Table A1. Association between ED visit volume and Telemedicine use among New England EDs, stratified by urban status

| Telemedicine Use Among Rural EDs (n=21) | | |
| --- | --- | --- |
|  | Rural Telemedicine Non-Users n=6 | Rural Telemedicine Users  n=15 |
| Annual total ED visits |  |  |
| <20,000 | 6 (100%) | 13 (87%) |
| 20,000-39,999 | 0 (0) | 2 (13%) |
| 40,000-59,999 | 0 (0) | 0 (0) |
| >60,000 | 0 (0) | 0 (0) |
| Telemedicine Use Among Urban EDs (n=148) | | |
| Annual total ED visits |  |  |
| <20,000 | 19 (23%) | 16 (24%) |
| 20,000-39,999 | 22 (27%) | 29 (43%) |
| 40,000-59,999 | 21 (26%) | 16 (24%) |
| >60,000 | 19 (23%) | 6 (9%) |

Table A2. Multivariable logistic regression models for telemedicine use among New England EDs


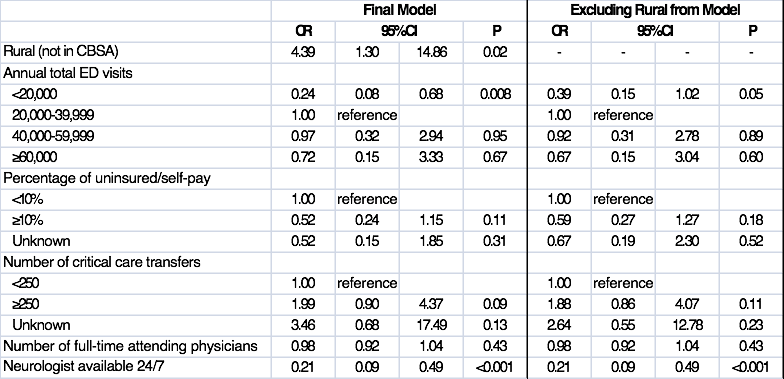

Supplement: Supplementary file 3 [file wjem-18-1055-s003.docx]
